# The Effects of Pregestational Overweight and Obesity on Maternal Lipidome in Pregnancy: Implications for Newborns’ Characteristics

**DOI:** 10.3390/ijms25137449

**Published:** 2024-07-07

**Authors:** Minja Derikonjic, Marija Saric Matutinovic, Sandra Vladimirov Sopic, Tamara Antonic, Aleksandra Stefanovic, Jelena Vekic, Daniela Ardalic, Milica Miljkovic-Trailovic, Marko Stankovic, Tamara Gojkovic, Jasmina Ivanisevic, Jelena Munjas, Snezana Jovicic, Zeljko Mikovic, Aleksandra Zeljkovic

**Affiliations:** 1 Department of Medical Biochemistry, University of Belgrade-Faculty of Pharmacy, Vojvode Stepe 450, 11000 Belgrade, Serbia; minja.derikonjic@pharmacy.bg.ac.rs (M.D.); marija.saric@pharmacy.bg.ac.rs (M.S.M.); sandra.vladimirov@pharmacy.bg.ac.rs (S.V.S.); antonic.tamara93@gmail.com (T.A.); alex@pharmacy.bg.ac.rs (A.S.); jelena.vekic@pharmacy.bg.ac.rs (J.V.); milica.miljkovic@pharmacy.bg.ac.rs (M.M.-T.); tamara.gojkovic@pharmacy.bg.ac.rs (T.G.); jasmina.ivanisevic@pharmacy.bg.ac.rs (J.I.); jelena.munjas@pharmacy.bg.ac.rs (J.M.); snezana.jovicic@pharmacy.bg.ac.rs (S.J.); 2 Gynecology and Obstetrics Clinic Narodni Front, Kraljice Natalije 62, 11000 Belgrade, Serbia; danielaardalic@gmail.com (D.A.); drmare84@hotmail.com (M.S.); mikovic.zeljko@gakfront.org (Z.M.)

**Keywords:** pregestational BMI, pregnancy, obesity, lipidome, neonatal outcome

## Abstract

Obesity is an important risk factor for the development of pregnancy complications. We investigated the effects of pregestational overweight and obesity on maternal lipidome during pregnancy and on newborns’ characteristics. The study encompassed 131 pregnant women, 99 with pre-pregnancy body mass index (BMI) < 25 kg/m^2^ and 32 with BMI ≥ 25 kg/m^2^. Maternal lipid status parameters, plasma markers of cholesterol synthesis and absorption and sphingolipids were determined in each trimester. Data on neonatal height, weight and APGAR scores were assessed. The results showed a higher prevalence (*p* < 0.05) of pregnancy and childbirth complications among the participants with elevated pregestational BMI. Levels of total cholesterol, HDL-cholesterol (*p* < 0.05) and LDL-cholesterol (*p* < 0.01) were significantly lower, and concentrations of triglycerides were higher (*p* < 0.05) in women with increased pre-gestational BMI. Lower concentrations of the cholesterol synthesis marker, desmosterol, in the 2nd trimester (*p* < 0.01) and the cholesterol absorption marker, campesterol, in each trimester (*p* < 0.01, *p* < 0.05, *p* < 0.01, respectively) were also found in this group. Markers of maternal cholesterol synthesis were in positive correlation with neonatal APGAR scores in the group of mothers with healthy pre-pregnancy weight but in negative correlation in the overweight/obese group. Our results indicate that gestational adaptations of maternal lipidome depend on her pregestational nutritional status and that such changes may affect neonatal outcomes.

## 1. Introduction

Metabolic, physical and hormonal changes that occur in the mother’s body during pregnancy are aimed to provide the fetus with nutrients and to prepare the mother for childbirth and breastfeeding. Although physiological, these metabolic alterations may lay the groundwork for the development of complications, such as gestational diabetes, hypertension, preeclampsia, or premature birth, and all of these can have negative consequences for both the mother and the child [1,2].

One of the main risk factors for the occurrence of pregnancy complications is obesity [3]. According to the World Health Organization data, overweight (body mass index; BMI > 25 kg/m^2^) and obesity (BMI > 30 kg/m^2^) affect about 60% of European adults. Of particular concern is the fact that there is an increase in the prevalence of obesity in women of reproductive age in many European countries [4]. While the impact of excessive gestational weight gain on pregnancy outcomes is well explored [5,6,7], fewer studies have examined the association of pre-pregnancy overweight and obesity with the development of pregnancy and delivery complications. Available data show that the prevalence of pregestational obesity in Europe is between 26.8% and 54% [8], although only a few European countries systematically publish reports on pre-pregnancy BMI [9], which might be one of the reasons for the scant investigation of this risk factor.

Changes in lipid metabolism are inherent to pregnancy [10]. Pregnancy-associated lipid profile changes develop due to increased lipolysis, the release of fatty acids from adipose tissue, and consequent increased hepatic synthesis of very low-density lipoprotein (VLDL) [11]. However, the question arises as to how increased pre-pregnancy BMI affects changes in lipid parameters during pregnancy, as well as pregnancy outcomes. So far, a small number of studies have dealt with this topic, and the results are inconsistent [12,13].

Although the determination of a routine lipid profile can provide information on dyslipidemia in pregnancy, novel methods of lipidomic analysis offer more in-depth insights into changes in maternal lipidome. Non-cholesterol sterols (NCSs) can be used as biomarkers of cholesterol synthesis and absorption, considering that they follow the same metabolic routes as cholesterol itself [14,15]. Given that cholesterol homeostasis is disturbed in obesity [16] and that inadequate cholesterol supply might have detrimental effects on fetal growth [17], it would be important to explore changes in cholesterol balance in relation to pre-pregnancy obesity.

Sphingolipids also have a physiological role in pregnancy but can be associated with the development of complications as well [18]. They are a structural component of biological membranes and participate in intercellular signaling as secondary messengers. One of the most studied sphingolipids is sphingosine-1-phosphate (S1P), which is bound to high-density lipoprotein (HDL) particles via apolipoprotein M and exhibits anti-atherosclerotic effects [18]. As obesity is generally accompanied by the reduced production of HDL particles, S1P will be more bound to albumin in the blood, which reduces its protective effect [19]. On the other hand, ceramides C16 (Cer C16:0) and C24 (Cer C24:0), also present on HDL particles, have proapoptotic and cytotoxic effects [18,20]. However, the sphingolipid profile in pregnancy accompanied by obesity has been scarcely explored.

Obesity before and during pregnancy can not only cause adverse effects for the mother but can also affect the newborn. Research shows that pregestational obesity can significantly contribute to the occurrence of neonatal macrosomia [21]. Additionally, recent studies have reported an increased risk of developing obesity in children whose mothers had increased BMI before pregnancy [22], as well as in children who did not have adequate intrauterine development [23]. Childhood obesity is known to be associated with dyslipidemia, which increases the risk of developing cardiometabolic diseases in later life [24]. Thus, the exploration of mutual relationships between maternal pregestational body weight, subsequent lipidome changes and neonatal outcomes might provide a novel look at metabolic adaptations aimed at ensuring a healthy pregnancy.

The aim of this study was to examine the link between pre-pregnancy BMI and changes in lipidome biomarkers during pregnancy. Additionally, we investigated whether there is an association between changes in lipidome parameters in pregnant women and the characteristics of newborns.

## 2. Results

Table 1 contains data on the general characteristics of pregnant women who were stratified according to pre-pregnancy BMI. There were no significant differences in age and pregestational smoking habits. Similarly, the prevalence of insulin resistance, hypothyroidism and thrombophilia did not differ among the groups. In contrast, pregestational hypertension was present only in the group with elevated BMI. Despite differences in pre-pregnancy BMI, the groups were comparable based on pregnancy weight gain. However, the prevalence of pregnancy and delivery complications was significantly higher among participants with elevated pregestational BMI. Although there were no differences in anthropometric measures of the offspring, we found lower values of 1 min and 5 min APGAR scores, as well as a higher prevalence of APGAR scores lower than 9 in newborns of mothers with higher pre-pregnancy BMI (Table 2).

Comparison of routine lipid status parameters between the groups (Table 3) revealed significantly higher TC concentrations, measured during the 3rd trimester, in the group of pregnant women with pregestational BMI < 25 kg/m^2^. In contrast, levels of TG in those subjects were consistently lower across trimesters, when compared with the high BMI group. We found higher concentrations of HDL-C in women with healthy pregestational weight in each trimester, although statistical significance was reached only in the 3rd trimester. Concentrations of LDL-C, measured in the 3rd trimester, were significantly higher in pregnant women who started their pregnancies with healthy weight.

Next, we explored lipidome characteristics in both groups across trimesters of pregnancy (Table 4). Regarding cholesterol synthesis markers, we found significantly higher desmosterol levels in the 2nd and 3rd trimesters in the group with BMI < 25 kg/m^2^. Concentrations of the cholesterol absorption marker, campesterol, were higher in women with healthy pre-pregnancy weight when measured in each trimester. A similar trend was observed for campesterol levels in the HDL fraction of plasma, with statistical significance reached in the 1st and 2nd trimesters. We found no differences in sphingolipid profiles among the examined groups, except for significantly higher SM concentrations in the 2nd and 3rd trimesters in women who were lean prior to gestation.

Correlation analysis in the group of women who were overweight or obese at the beginning of pregnancy (Table 5) showed a significant positive association between TG levels in the 2nd and 3rd trimesters and newborns’ weight and length. Additionally, TG concentrations in each trimester were negatively correlated with 5 min APGAR scores. HDL-C levels in the 3rd trimester were positively correlated, while desmosterol measured in the 1st and the 2nd trimester correlated negatively with APGAR scores. 7-dehydrocholesterol measured during the 3rd trimester in both whole plasma and HDL fraction of plasma correlated negatively with anthropometric characteristics of neonates. Cer24 and Cer16 values across trimesters positively correlated with newborn weight and height, while Cer24 measured in the 1st trimester was in negative correlation with the APGAR score. Concentrations of SPH, S1P and SPA-1P in the 2nd trimester positively correlated with APGAR scores, whereas SPA measured in the 1st trimester was in negative correlation with these scores. S1P levels measured in the 2nd trimester were in negative correlation with neonatal length.

Analogous analysis in the group of lean women prior to gestation (Table 6) demonstrated a significant positive correlation between TC in the 3rd trimester and newborn’s length. Concentrations of campesterol in whole plasma, as well as desmosterol and 7-dehydrocholesterol in HDL fraction of plasma during the 2nd trimester positively correlated with 5 min APGAR scores. SPA and S1P levels in the 3rd trimester positively correlated with head circumference and length of newborns, respectively. Finally, SPA-1P in the 1st trimester was negatively correlated with newborns’ weight.

## 3. Discussion

In this study, we have shown significant differences in the lipidome of pregnant women with normal and increased pre-pregnancy BMI. In addition, intriguing relationships were found among specific maternal lipidome components and characteristics of neonates at delivery.

It was previously reported that pregestational overweight and obesity can increase the risk of the development of pregnancy complications [25]. Accordingly, our results revealed a higher prevalence of pregnancy and delivery complications in women with elevated pregestational BMI, even though there were no differences in pregnancy weight gain among the observed groups. Similarly, APGAR score values were lower in newborns of mothers who were overweight or obese in the periconceptional period. However, the metabolic basis for such an association was not sufficiently explored. The development of dyslipidemia is a common point between obesity and pregnancy. Alterations in lipid status are intrinsic to pregnancy and aimed to ensure an adequate fetal energy supply, but herein we observed that pre-pregnancy obesity and overweight can modify otherwise typical lipid profile changes; namely, concentrations of TC, LDL-C and HDL-C were significantly lower by the end of pregnancy in women with elevated pregestational BMI, while the levels of TG were consistently higher in this group. Similar results were obtained in other studies [13,26,27]. Thus, it seems that a pregnancy-associated rise in maternal cholesterol is less prominent in pre-pregnancy obese and overweight women. More in-depth lipidome analysis of NCSs revealed that the cholesterol synthesis marker, desmosterol, and the cholesterol absorption marker, campesterol, were consistently lower in women with increased pregestational BMI, suggesting diminished capacities of cholesterol synthesis and absorption in this group. Given that cholesterol is a prerequisite for fetal growth, we can hypothesize that a less favorable lipid profile before pregnancy in overweight and obese women might be associated with an inadequate adaptation of maternal cholesterol metabolism during pregnancy. To the best of our knowledge, alterations in maternal cholesterol homeostasis in the context of pre-pregnancy obesity have been scarcely explored so far. Thus, our preliminary findings might incite further investigations in this direction. A specific feature of pregnancy-induced dyslipidemia is a slight, but consistent elevation of HDL-C [28]. Apart from the well-known atheroprotective roles of HDL, the beneficial effects of these particles have been demonstrated in pregnancy as well; namely, HDL is involved in fetal cholesterol supply and maintaining placental vascular function [29]. Our finding of decreased gestational HDL-C levels in pre-pregnancy overweight and obese women implies its reduced protective properties. Moreover, we found decreased levels of campesterol in HDL in this group. Appreciating that HDL-campesterol levels could be seen as a measure of intestinal HDL synthesis [30], the obtained results suggest compromised HDL formation in case of pre-pregnancy overweight and obesity. Interestingly, significant changes in HDL-campesterol were observed at the beginning of pregnancy, implying early development of unfavorable lipidome alterations in pre-pregnancy obese and overweight women.

Although the determination of routine lipid profile is a simple and easily accessible test for examining the lipidome differences between obese pregnant women and those with normal BMI, recent research has focused on changes in other lipid moieties. In addition to cholesterol, sphingolipids are important constituents of lipoproteins. We observed an increase in the concentration of SM in both groups of pregnant women during the three trimesters, and these results agree with previous studies of changes in SM during pregnancy [31]. On the other hand, our results showed that the levels of SM were significantly lower during the second and third trimesters in the group of pregnant women who had a BMI ≥ 25 kg/m^2^ before pregnancy. Although its roles in pregnancy are not entirely clear [17], it is known that SM is produced by the fetal lungs before surfactant synthesis begins [32]. Considering that SM is a binding component of LDL particles, but also a part of HDL particle composition [33], the obtained results confirmed profound derangements of lipoprotein particle metabolism in case of pregestational overweight and obesity.

We further examined correlations between the lipidome parameters in both groups of pregnant women and the characteristics of newborns. We observed a positive association between TG concentrations in women with increased pre-gestational BMI and the height and weight of newborns. Such results are consistent with the results of other studies [34,35,36]. The basis for this correlation lies in the role of free fatty acids, which the fetus receives through hydrolysis of the mother’s TG. TG are necessary for the fetus to develop, but they are also associated with the promotion of insulin resistance. Extensive lipolysis driven by poorly controlled insulin resistance contributes to the increase in fetal weight [36,37]. This correlation was not observed in the group of women with normal pregestational BMI, which indicates that newborns of obese women have a higher risk of developing macrosomia. This assumption was confirmed by the latest research conducted by Grobeisen-Dukue and colleagues [38]. Additionally, considering that the level of TG is negatively correlated with the APGAR score and that a lower APGAR score is associated with the occurrence of complications in the child [39], this is another indicator of the negative impact of maternal obesity before pregnancy on neonatal outcomes.

Our results indicate a positive correlation between ceramide concentrations and neonatal weight in the group of mothers who had an increased pre-pregnancy BMI. Although we did not find any studies examining these specific correlations, recent research has shown an association between obesity and high ceramide levels. Ceramide metabolism in obesity is extremely complex and still insufficiently investigated, but it is known that obesity is accompanied by the accumulation of these sphingolipids [40]. Additionally, it has been shown that high ceramide concentration in pregnant women is associated with the development of complications such as gestational diabetes mellitus and preeclampsia [41,42].

When it comes to S1P, a negative correlation was observed between this sphingolipid and the length of newborns of mothers who had an increased BMI before pregnancy, while this correlation was positive in the group of lean women. Given that S1P is a constituent of HDL particles, we can explain the observed findings by the previously described roles of HDL in pregnancy; namely, the formation of dysfunctional HDL particles in pregnant women with elevated pre-pregnancy BMI could be responsible for the negative link between S1P and neonatal length. It was previously postulated that the functional properties of S1P strongly depend on its association with HDL [19]. Therefore, it is possible that the observed negative correlation could be a reflection of impaired functionality of the HDL–S1P axis in obese pregnant women. A compromised functionality of HDL particles, which serve as carriers of S1P, might influence this sphingolipid’s properties as well. Thus, obesity, via disturbed HDL structure and function, might trigger the lowering of S1P functional capacity. On the other hand, a positive association between maternal S1P and neonatal length in the case of normal maternal body weight prior to pregnancy might confirm previously reported beneficial effects of S1P on fetoplacental vasculature [43] and thus on neonatal development, if proper HDL metabolism is present. Indeed, it has been shown that during early pregnancy, S1P stimulates placental angiogenesis through its receptors and plays a key role in the preservation of the endothelial barrier [44].

Intriguing correlations were found for lipidome parameters and APGAR scores. In the group of pre-pregnancy overweight and obese women, neonatal APGAR scores were in negative correlation with TG levels and in positive correlation with HDL concentrations. The observed associations are in line with the previously mentioned detrimental effects of TG and protective effects of HDL on the entire fetoplacental unit. The majority of analyzed sphingolipid species were in positive correlation with APGAR scores. Sphingolipids in pregnancy are mainly explored as biomarkers of preeclampsia. Studies have shown that in this state, concentrations of ceramide, SPH, S1P and SM in maternal plasma increase [45]. However, data on their associations with neonatal characteristics are lacking. Of note, increased SPA and decreased SPA-1P levels were reported in umbilical cord vein samples of newborns of mothers with preeclampsia [46]. Since we also observed divergent associations between the mentioned sphingolipid species and neonatal outcomes, it is possible that they conversely affect neonatal development. It should also be mentioned that in our study, a higher frequency of complications was found in pre-pregnancy obese and overweight women. Taken altogether, we could hypothesize that alterations in specific sphingolipids may present a bridge between maternal obesity and unfavorable pregnancy outcomes, which should be explored further in large-scale prospective studies.

Significant findings in our study are the observed divergent correlations between cholesterol synthesis markers and APGAR scores in the two examined groups; namely, a positive correlation between NCSs and APGAR score was seen in neonates of mothers with pregestational BMI < 25 kg/m^2^, thus underlining the importance of cholesterol supply for a proper fetal and neonatal development. However, this association was negative for newborns delivered by pre-pregnancy overweight and obese mothers. These results imply that obesity-driven disturbances in cholesterol homeostasis exhibit unfavorable effects not only for the mother but also for the offspring. Due to all the above, the recommendations for the regulation of body weight before pregnancy, through a healthy diet and physical activity to prevent the occurrence of complications [47] are of paramount importance.

The observed associations between maternal lipidome changes and neonatal outcomes lay the groundwork for future studies aimed at elucidating corresponding molecular mechanisms. Epigenetic alterations in both mothers and offspring could be responsible for the relationships between maternal obesity, dyslipidemia and neonatal characteristics [48]. Therefore, future studies should be oriented towards the exploration of epigenetic traits in maternal and cord blood, as well as in the placenta, with respect to maternal pregestational and gestational weight. Additionally, follow-up of newborns of overweight and obese mothers would be very useful in elucidating the long-term health impacts of maternal obesity. Moreover, while herein the focus was on examining maternal lipidome, it should be emphasized that lipoprotein particles also include proteins in their composition. Therefore, further research could be directed toward investigating the impact of pregestational BMI on the proteome of pregnant women.

In conclusion, this study is one of the first to extensively examine the lipidome in pregnant women, considering their pre-pregnancy BMI. Our results imply that gestational adaptations of maternal lipidome depend on her pregestational nutritional status. Additionally, we showed that maternal lipidome changes are associated with basic characteristics of the offspring, primarily weight and APGAR score. Since maintaining proper lipid homeostasis in pregnancy is essential for both the mother and the child, research in this direction should be continued to fully elucidate the impact of periconceptual obesity on lipidome in pregnancy and its short-term and long-term consequences.

## 4. Materials and Methods

### 4.1. Patients

This study is part of a larger research project that originally enrolled 239 pregnant women whose pregnancies were monitored in the Obstetrics and Gynecology Clinic Narodni Front in Belgrade. The participants were recruited during their first medical check-up at the beginning of pregnancy. Exclusion criteria comprised multiparous pregnancy, any acute conditions at the time of medical examination, the presence of chronic liver and kidney diseases prior to pregnancy, as well as the use of any lipid-lowering medications. Existing pregestational medical conditions in the study cohort included insulin resistance (9 cases), hypothyroidism (8 cases), thrombophilia (11 cases) and hypertension (4 cases). All participants were followed throughout the entire pregnancy until delivery. Clinical and laboratory data were collected during regular medical follow-ups that were organized in each trimester. The control points, at which blood samples were taken, were scheduled in the 1st trimester (median: 13.2; interquartile range: 13.0–13.6 gestational weeks), the 2nd trimester (median: 22.3; interquartile range: 21.6–23.5 gestational weeks) and the 3rd trimester (median: 32.9; interquartile range: 32.0–33.4 gestational weeks). Neonatal data were collected from medical records taken at delivery. A total of 131 women attended all scheduled clinical and laboratory evaluations and formed the final study group.

In 89 cases, pregnancy and delivery were without any complications, while 42 women experienced one or multiple complications during pregnancy and/or at delivery, including gestational diabetes (11 cases), gestational hypertension (13 cases), pre-eclampsia (2 cases), preterm delivery (6 cases), prolonged delivery (5 cases), fetal asphyxia (1 case), oligohydramnios (8 cases), polyhydramnios (1 case) and morbidly adherent placenta (3 cases).

Body mass index (BMI) was defined as weight (kg)/height^2^ (m^2^). Pregnancy weight gain was recorded in each trimester. Excessive weight gain during pregnancy was estimated according to the recommendations given by the American College of Obstetricians and Gynecologists [49].

All participants signed an informed consent prior to enrollment. The entire study was designed and conducted in accordance with the ethical guidelines defined by the Helsinki Declaration and was approved by the local ethical committees.

### 4.2. Sampling and Methods

Blood samples were taken during each control examination. Blood was drawn into evacuated tubes after overnight fasting. Serum and plasma were separated, aliquoted, stored at −80 °C and thawed immediately before analysis.

Concentrations of lipid status parameters, total cholesterol (TC), triglycerides (TG) and HDL cholesterol (HDL-C) were determined using routine automated methods and commercial test reagents (Beckman Coulter, Brea, CA, USA). Low-density lipoprotein cholesterol (LDL-C) levels were estimated using the Friedwald equation.

### 4.3. Determination of NCSs

Quantification of NCSs in plasma and HDL fractions, which include cholesterol synthesis (desmosterol and lathosterol) and absorption (campesterol and β-sitosterol) markers, was performed using our previously developed HPLC/MS-MS method [50]. HPLC-grade analytical standards for quantifying desmosterol, lathosterol, campesterol and β-sitosterol were purchased from Supelco (Bellefonte, PA, USA). Deuterated internal standard (IS) d6-cholesterol (HPLC-grade) and cholesterol standard (purity of ≥99%) for constructing the calibration curve were obtained from Sigma-Aldrich (St. Louis, MO, USA). For separation of the HDL fraction, a commercial precipitating reagent manufactured by Bio-Systems (Costa Brava, Barcelona, Spain) was utilized.

For total NCS analysis, 100 μL of plasma was mixed with dried IS (50 μL, d6-cholesterol, 1 mg/mL), followed by the addition of 1 mL of 2% ethanolic KOH and incubation at 45 °C for 30 min. Extraction was performed by adding an extraction mixture of n-hexane/water (4:1), followed by centrifugation at 1500× *g* for 5 min and collection of the upper layers in a clean glass tube. This step was repeated three times after which excess KOH was removed using 4 mL of HPLC-grade deionized water. The final extract was dried and reconstituted in 20 μL of HPLC-grade methanol. For the determination of NCSs in HDL, an ApoB-depleted plasma fraction was utilized. In this procedure, 200 μL of plasma was mixed with 500 μL of ApoB-precipitation reagent. After vortexing and a 10 min incubation, the mixture was centrifuged for an additional 10 min (6000 rpm). Then, 650 μL of HDL supernatant was added to a conical glass tube containing dried IS and processed using the same sample preparation method, as described above.

We used the Porochell 120 EC column (150 × 4.6 mm × 2.7 mm) from Agilent Technologies (USA) for the chromatographic separation of NCSs. Isocratic elution was performed using acetonitrile:methanol:water with 0.1% formic acid (80:18:2, *v*/*v*), at a mobile phase flow rate of 0.6 mL/min and a column temperature of 30 °C. The m/z transitions for each analyte were specified. Quantification was carried out using multiple reaction monitoring (MRM) on the Agilent 6420 triple quad mass spectrometer equipped with an APCI ion source (Agilent Technologies, Santa Clara, CA, USA).

### 4.4. Determination of Sphingolipid Species

HPLC-grade analytical standards for quantification of sphingosine (SPH), sphinganine (SPA), S1P, ceramide C16:0 (Cer C16:0) and ceramide C24:0 (Cer C24:0) were obtained from Avanti Polar Lipids (Birmingham, AL, USA), while sphinganine-1-phosphate (SPA-1P) and sphingomyelin C16:0 (SM) were purchased from Cayman Chemical (Ann Arbor, MI, USA). We used SPH d17, SPH-1P d17, ceramide C17:0 (Cer C17:0) (Avanti Polar Lipids, Birmingham, AL, USA) and sphingomyelin C17:0 (SM C17:0) (Cayman Chemical, Ann Arbor, MI, USA) as internal standards (IS).

Quantification of specific sphingolipids was carried out using the HPLC-MS/MS procedure, after a preparatory sample extraction step. Initially, lipids were extracted from plasma using a liquid–liquid extraction method. For total sphingolipid analysis, a 50 µL aliquot of plasma was added to a glass vial containing a mixture of dried IS. Extraction was carried out by adding 2 mL of a methanol:chloroform mixture (2:1 ratio) with 0.1% trifluoroacetic acid to each vial and vortexed for 30 s. Subsequently, 0.67 mL of chloroform was added and the vials were mixed again for 30 s. Afterward, 1.15 mL of HPLC-grade water was added to form a mixture of methanol, chloroform and water at a ratio of 1:1:0.9. Finally, the samples were vortexed for 30 s and centrifuged for 20 min at 2000× *g*. The lower chloroform layer was transferred to a clean glass tube. The final extract was dried and reconstituted in 30 µL of HPLC-grade methanol. The concentrated specimen underwent an additional 30 s of vortexing and centrifugation at 1500× *g* for 10 min before injection into the column.

We used a Zorbax Eclipse Plus C8 column (4.6 × 150 mm, 5 µm) from Agilent Technologies (Santa Clara, CA, USA) for chromatographic separation of sphingolipids. Gradient elution (solvent A (1 mM ammonium formate in methanol with 0.2% formic acid) and solvent B (2 mM ammonium formate in water with 0.2% formic acid)) was performed, with the initial condition set to 80% A and 20% B and a subsequent linear gradient to 90% A for 10.6 min. This composition was maintained for 6 min, followed by a linear gradient to 99% A over 66 min and a linear gradient to 80% A from 66 to 68 min. The final condition (80% A and 20% B) was maintained for 7 min. The mobile phase flow rate was constant at 0.5 mL/min. The injection sample volume was 15 µL.

Quantification was performed using an MRM mode of data acquisition on the Agilent 6420 triple quad with an ESI ion source (Agilent Technologies, Santa Clara, CA, USA). Source conditions were as follows: gas and vaporizer temperature of 340 °C and 250 °C, respectively, nebulizer pressure at 20 psi, a gas flow rate of 12 L/min, a positive capillary voltage of 4500 V and a negative capillary voltage of 4000 V.

### 4.5. Statistical Analysis

The normality of data distribution was tested using the Kolmogorov–Smirnov test and the Shapiro–Wilk test. Continuous variables that followed normal distribution were presented as mean ± standard deviation and compared using the Student’s t-test. Asymmetrically distributed variables were given as median (interquartile range) and compared using the Mann–Whitney U test. Categorical variables were presented as relative frequencies and comparisons were made using the Chi-squared test. Significant correlations were explored using Spearman’s correlation analysis.

Statistical tests were considered significant if *p* < 0.05. Statistical analyses were performed using the statistical package PASW Statistics 18 (IBM, Armonk, NY, USA).

## Figures and Tables

**Table 1:** General characteristics of study participants grouped according to pre-pregnancy BMI.

	BMI < 25 kg/m^2^	BMI ≥ 25 kg/m^2^	*p*
N	99	32	
Age (years)	32.07 ± 5.51	32.13 ± 5.36	0.961
Pregestational BMI (kg/m^2^)	20.93 ± 2.15	28.73 ± 3.36	<0.001
Smoking before pregnancy (%)	28.87	34.38	0.556
Insulin resistance (%)	7.07	6.25	0.873
Hypothyroidism (%)	5.05	9.38	0.374
Thrombophilia (%)	10.10	3.13	0.216
Hypertension (%)	0	12.50	<0.001
Pregnancy weight gain (kg) *	18.00 (12.00–23.00)	19.50 (11.00–27.25)	0.656
Excessive pregnancy weight gain (%) #	53.68	51.72	0.853
Pregnancy/delivery complications (%) #	25.25	53.13	0.003

Data are presented as mean ± standard deviation and compared using the Student’s *t*-test. * Data are presented as median (interquartile range) and compared using the Mann–Whitney U test. # Data are presented as relative frequencies and compared using the Chi-squared test.

**Table 2:** Neonatal outcomes based on maternal pre-pregnancy BMI.

Parameter	BMI < 25 kg/m^2^	BMI ≥ 25 kg/m^2^	*p*
Newborn weight (g)	3410.00 (3135.00–3690.00)	3250.00 (3070.00–3817.15)	0.830
Newborn length (cm)	51.00 (50.00–52.00)	51.00 (50.00–52.00)	0.578
Newborn head circumference (cm)	35.00 (34.00–36.00)	35.00 (34.00–36.00)	0.533
1 min APGAR score	9.00 (9.00–9.00)	9.00 (8.25–9.00)	0.002
5 min APGAR score	10.00 (10.00–10.00)	10.00 (9.25–10.00)	0.002
1 min APGAR score below 9 (%) #	6.12	25.00	0.029
5 min APGAR score below 9 (%) #	1.02	9.38	0.016

Data are presented as median (interquartile range) and compared using the Mann–Whitney U test. # Data are presented as relative frequencies and compared using the Chi-squared test.

**Table 3:** Comparison of routine lipid parameters between the groups across trimesters of pregnancy.

Parameter	Trimester	BMI < 25 kg/m^2^	BMI ≥ 25 kg/m^2^	*p*
TC (mmol/L)	T1	5.34 ± 0.97	5.35 ± 1.00	0.948
	T2	6.81 ± 1.34	6.42 ± 1.38	0.170
	T3	7.33 ± 1.50	6.45 ± 1.57	0.007
TG * (mmol/L)	T1	1.18 (0.96–1.49)	1.48 (1.10–1.76)	0.038
	T2	1.76 (1.40–2.16)	2.10 (1.69–2.64)	0.011
	T3	2.35 (1.97–2.95)	2.83 (2.48–3.20)	0.027
HDL-C (mmol/L)	T1	1.75 ± 0.33	1.62 ± 0.32	0.052
	T2	1.89 ± 0.33	1.80 ± 0.36	0.245
	T3	1.83 ± 0.31	1.70 ± 0.30	0.038
LDL-C (mmol/L)	T1	3.00 ± 0.85	3.00 ± 0.82	0.986
	T2	4.07 ± 1.16	3.62 ± 1.17	0.068
	T3	4.31 ± 1.25	3.42 ± 1.39	0.001

Data are presented as mean ± standard deviation and compared using the Student’s *t*-test. * Data are presented as median (interquartile range) and compared using the Mann–Whitney U test.

**Table 4:** Differences in lipidome biomarkers between the groups across trimesters of pregnancy.

Parameter	T1			T2			T3		
	BMI < 25 kg/m^2^	BMI ≥ 25 kg/m^2^	*p*	BMI < 25 kg/m^2^	BMI ≥ 25 kg/m^2^	*p*	BMI < 25 kg/m^2^	BMI ≥ 25 kg/m^2^	*p*
Desmosterol (μmol/L)	2.58 (2.06–3.14)	2.59 (2.17–3.39)	0.962	3.62 (2.86–4.47)	2.88 (2.32–3.66)	0.003	4.62 (3.69–6.09)	4.00 (3.40–4.72)	0.025
7-Dehydrochole-sterol (umol/L)	1.75 (1.44–2.40)	1.95 (1.57–2.46)	0.210	2.14 (1.74–2.67)	1.92 (1.56–2.70)	0.159	2.39 (1.93–3.03)	2.38 (1.93–2.95)	0.420
Lathosterol (μmol/L)	10.53 (7.53–15.50)	14.00 (7.43–17.41)	0.340	18.41 (13.27–24.88)	17.23 (12.09–22.00)	0.271	23.20 (17.57–31.98)	20.48 (16.47–29.23)	0.279
Campesterol (μmol/L)	5.12 (3.85–6.59)	3.58 (2.43–5.00)	0.001	5.82 (4.61–7.26)	4.44 (3.48–6.14)	0.020	5.77 (4.16–6.97)	4.23 (3.53–5.22)	0.003
β-Sitosterol (μmol/L)	22.15 (15.40–27.91)	24.81 (15.90–29.74)	0.627	20.11 (15.51–27.17)	24.73 (15.09–27.08)	0.424	19.24 (14.83–25.91)	22.96 (16.41–26.12)	0.217
Desmosterol in HDL	0.26 (0.20–0.31)	0.25 (0.17–0.32)	0.886	0.27 (0.23–0.33)	0.31 (0.22–0.40)	0.199	0.32 (0.24–0.42)	0.32 (0.24–0.40)	0.927
7-Dehydrochole-sterol in HDL (umol/L)	0.39 (0.33–0.47)	0.39 (0.31–0.45)	0.835	0.40 (0.33–0.51)	0.42 (0.37–0.51)	0.346	0.41 (0.34–0.48)	0.38 (0.35–0.47)	0.477
Lathosterol in HDL (μmol/L)	1.07 (0.74–1.61)	1.33 (0.87–1.81)	0.135	1.29 (0.87–1.72)	1.60 (1.94–2.11)	0.053	1.37 (0.97–2.03)	1.34 (0.96–2.22)	0.372
Campesterol in HDL (μmol/L)	0.53 (0.31–0.77)	0.39 (0.19–0.49)	0.008	0.50 (0.37–0.71)	0.35 (0.67–0.58)	0.013	0.43 (0.31–0.63)	0.37 (0.24–0.50)	0.135
β-Sitosterol in HDL (μmol/L)	5.49 (4.11–7.90)	4.92 (3.5–6.32)	0.082	5.48 (4.27–7.70)	4.88 (3.50–5.94)	0.065	5.07 (4.28–8.45)	5.05 (3.78–5.89)	0.188
CerC24 (μmol/L)	1.27 (0.73–1.98)	1.55 (0.58–2.18)	0.816	1.94 (1.21–2.81)	1.95 (1.50–2.71)	0.676	2.86 (1.89–4.16)	3.65 (2.67–3.85)	0.732
CerC16 (μmol/L)	0.38 (0.30–0.45)	0.40 (0.33–0.63)	0.645	0.38 (0.30–0.46)	0.48 (0.39–0.60)	0.087	0.46 (0.35–0.60)	0.59 (0.45–0.70)	0.626
SPH (nmol/L)	52.60 (40.21–65.79)	58.23 (42.84–79.24)	0.764	54.77 (45.67–74.24)	77.34 (50.16–126.89)	0.464	62.73 (50.49–83.80)	61.30 (47.57–82.88)	0.766
SPA (nmol/L)	15.05 (11.75–18.29)	17.71 (13.89–21.97)	0.801	17.82 (13.83–20.97)	17.44 (14.82–24.33)	0.848	21.72 (17.83–26.86)	19.26 (13.93–25.40)	0.210
S1P (nmol/L)	477.84 (350.11–728.45)	433.28 (351.54–1106.85)	0.396	505.94 (414.77–887.91)	528.03 (348.89–685.42)	0.182	625.00 (477.51–971.49)	515.91 (404.70–817.43)	0.130
SPA-1P (nmol/L)	101.56 (71.17–153.26)	74.74 (48.81–147.91)	0.736	105.72 (74.72–163.73)	113.44 (32.77–180.61)	0.420	100.06 (56.22–149.25)	89.88 (55.68–185.38)	0.371
SM (μmol/L)	319.77 (294.09–351.36)	298.64 (284.40–335.74)	0.199	368.73 (333.59–395.35)	334.30 (308.79–366.77)	0.016	383.86 (356.21–426.68)	346.12 (311.41–376.40)	0.012

Data are presented as median (interquartile range) and compared using the Mann–Whitney U test.

**Table 5:** Correlations between newborn’s characteristics and maternal lipidome parameters in prepregnancy overweight and obese women.

	Newborn Weight (g)	Newborn Length (cm)	Newborn Head Circumference (cm)	5 min APGAR Score
TG (mmol/L) T1				−0.591 ***
TG (mmol/L) T2	0.474 **			−0.467 **
TG (mmol/L) T3	0.376 *	0.405 *		−0.382 *
HDL-C (mmol/L) T3				0.402 *
Desmosterol (umol/L) T1				−0.410 *
Desmosterol (umol/L) T2				−0.460 *
7-Dehydrocholesterol (umol/L) T3	−0.446 *	−0.479 **		
7-Dehydrocholesterol in HDL (μmol/L) T3		−0.485 **		
CerC24 (μmol/L) T1	0.397 *	0.398 *		−0.478 **
CerC24 (μmol/L) T3	0.382 *	0.390 *		
CerC16 (μmol/L) T1	0.353 *			
CerC16 (μmol/L) T2	0.401 *			
CerC16 (μmol/L) T3	0.449 *	0.368 *		
SPH (nmol/L) T2				0.395 *
SPA (nmol/L) T1				−0.526 **
S1P (nmol/L) T2		−0.396 *		0.402 *
SPA-1P (nmol/L) T2				0.650 **

* *p* < 0.05; ** *p* < 0.01; *** *p* < 0.001.

**Table 6:** Correlations between newborn’s characteristics and maternal lipidome parameters in women with pre-pregnancy BMI < 25 kg/m^2^.

	Newborn Weight (g)	Newborn Length (cm)	Newborn Head Circumference (cm)	5 min APGAR Score
Cholesterol (mmol/L) T3		0.213 *		
Campesterol (μmol/L) T2				0.211 *
Desmosterol in HDL (µmol/L) T2				0.326 **
7-Dehydrocholesterol in HDL (μmol/L) T2				0.281 **
SPA (nmol/L) T3			0.214 *	
S1P (nmol/L) T3		0.204 *		
SPA-1P (nmol/L) T1	−0.216 *			

* *p* < 0.05; ** *p* < 0.01.

## Data Availability

The data presented in this study are available on request from the corresponding author. The data are not publicly available due to privacy and ethics considerations.
